# Age-based Human Influenza A Virus (H5N1) Infection Patterns, Egypt

**DOI:** 10.3201/eid11601.090560

**Published:** 2010-01

**Authors:** Alan Schroedl

**Affiliations:** Anthrometrix Corporation, Salt Lake City, Utah, USA

**Keywords:** Influenza A virus, H5N1 subtype, viruses, infection patterns, Egypt, letter

**To the Editor:** In April 2009, a representative of the World Health Organization in Cairo voiced concern about the changing age-based pattern of human influenza A virus (H5N1) infection in Egypt (*1*). From March 2006 through July 2009, a total of 83 persons in Egypt were confirmed to have human influenza A (H5N1); the patients’ ages ranged from >1 year to 75 years (*2*). However, from December 2008 through July 2009 in Egypt, 28 of 32 human infections were in children <8 years of age.

The frequency of human influenza A virus (H5N1) infections parallels the pattern for seasonal influenza. Thus, for analytical purposes, virus subtype H5N1 infections in Egypt can be grouped into 12-month periods, beginning with August of 1 year and ending in July of the following year. The results for 1-way analysis of variance indicate that the age at time of virus subtype H5N1 infection in Egypt differs significantly among these 4 periods (Kruskal–Wallis test statistic = 20.732, p<0.0004 ).

Further analysis shows that persons infected from August 1, 2008 through July 31, 2009, were much younger than those infected in the preceding 12-month period (Mann-Whitney U test statistic = 328.500, p<0.001). The median age of the 12 confirmed case-patients from August 1, 2007, through July 31, 2008, was 23.5 years, but the median age of the 33 confirmed case-patients from August 1, 2008, through July 31, 2009, was 3.0 years. The Table shows the distribution of case-patients by age group, the median age of each group, and the case-fatality ratio (CFR) for the 4 seasonal 12-month periods.

**Table:** Age groups, median ages, and case-fatality ratios for influenza A (H5N1) case-patients, by influenza season, Egypt*

Influenza season, August–July	No. case-patients by age group, y						Total no. case-patients	Median age, y	Case-fatality ratio
	1–8	9–20	21–30	31–40	41–74	>75			
2005–06	4	4	3	2	0	1	14	18.0	42.8
2006–07	13	5	4	2	0	0	24	6.5	37.5
2007–08	3	2	5	1	1	0	12	23.5	58.3
2008–09	28	1	1	3	0	0	33	3.0	15.1
Total	48	12	13	8	1	1	83	—	—

*Age at date of clinical sign onset; data from the World Health Organization (*2*).

This recent rise of subtype H5N1 influenza cases among children represents a major change in the pattern of human influenza A virus (H5N1) infections in Egypt compared with the pattern for earlier influenza seasons. Confirmation reports by the World Health Organization generally indicate associations with dead and sick poultry for these recent cases among children. The cultural patterns and customs of poultry husbandry have not changed in Egypt since the first human cases of influenza A (H5N1) were confirmed in 2006; thus, it is not clear why more children have been infected since December 2008. One explanation may be the increased recognition of the clinical signs of nonfatal influenza A (H5N1) among children and increased confirmation by laboratory testing. The lack of influenza A virus (H5N1) infection among the infected children’s parents and caregivers suggests that the virus is still not easily transmissible among humans in Egypt.

Not only has there been a recent increase in infections of influenza A (H5N1) among children, but there has also been a recent decline in deaths among confirmed infected persons. From 2006 through 2008, the annual CFR for influenza A (H5N1) in Egypt ranged from 36% to 55% (*3*). Since January 1, 2009, the CFR in Egypt has been 11%. The recent increases in infections among children coupled with a decrease in the CFR in the most recent 12-month period suggests that the strain of influenza A virus (H5N1) now circulating in Egypt may be becoming less virulent as it continues to spread among young children, a segment of the population that is highly vulnerable to influenza infections (*4*,*5*).
